# miR-31-5p Promotes Oxidative Stress and Vascular Smooth Muscle Cell Migration in Spontaneously Hypertensive Rats via Inhibiting FNDC5 Expression

**DOI:** 10.3390/biomedicines9081009

**Published:** 2021-08-13

**Authors:** Bing Zhou, Lu-Lu Wu, Fen Zheng, Nan Wu, Ai-Dong Chen, Hong Zhou, Jing-Yu Chen, Qi Chen, Yue-Hua Li, Yu-Ming Kang, Guo-Qing Zhu

**Affiliations:** 1 Key Laboratory of Targeted Intervention of Cardiovascular Disease, Collaborative Innovation Center for Cardiovascular Disease Translational Medicine, and Department of Physiology, Nanjing Medical University, Nanjing 211166, China; 579612zhoubing@wnmc.edu.cn (B.Z.); luluuu@njmu.edu.cn (L.-L.W.); fenzh@njmu.edu.cn (F.Z.); nanwu@njmu.edu.cn (N.W.); aidongchen@njmu.edu.cn (A.-D.C.); hongzhou@njmu.edu.cn (H.Z.); jychenjy@163.com (J.-Y.C.); 2 Department of Pathophysiology, Nanjing Medical University, Nanjing 211166, China; qichen@njmu.edu.cn (Q.C.); yhli@njmu.edu.cn (Y.-H.L.); 3 Cardiovascular Research Center, Department of Physiology and Pathophysiology, Xi’an Jiaotong University School of Medicine, Xi’an 710061, China; ykang@mail.xjtu.edu.cn

**Keywords:** miRNA, FNDC5, oxidative stress, cell migration, vascular smooth muscle cells, hypertension

## Abstract

Oxidative stress and the migration of vascular smooth muscle cells (VSMCs) are important for vascular remodeling in a variety of vascular diseases. miR-31-5p promotes cell migration in colorectal cancer cells but inhibits cell migration in renal cell carcinoma. However, whether miR-31-5p is involved in oxidative stress and VSMC migration remains unknown. This study shows the crucial roles of miR-31-5p in oxidative stress and VSMC migration, as well as underlying mechanisms. Experiments were carried out in primary VSMCs from aortic media of Wistar–Kyoto rats (WKY) and spontaneously hypertensive rats (SHR), as well as the A7r5 cell line. Oxidative stress was assessed by NADPH oxidase (NOX) expression, NOX activity, and reactive oxygen species (ROS) production. Cell migration was evaluated with a Boyden chamber assay and a wound healing assay. The miR-31-5p mimic and inhibitor promoted and attenuated oxidative stress and cell migration in the VSMCs of SHR, respectively. A dual-luciferase reporter assay indicated that miR-31-5p targeted the 3’UTR domain of FNDC5. The miR-31-5p level was raised and FNDC5 expression was reduced in the VSMCs of SHR compared with those of WKY. The miR-31-5p mimic reduced FNDC5 expression in the A7r5 cells and the VSMCs of both WKY and SHR, while the miR-31-5p inhibitor only increased FNDC5 expression in the VSMCs of SHR. Exogenous FNDC5 attenuated not only the oxidative stress and VSMC migration in SHR but also the roles of the miR-31-5p mimic in inducing oxidative stress and VSMC migration. These results indicate that miR-31-5p promotes oxidative stress and VSMC migration in SHR via inhibiting FNDC5 expression. The increased miR-31-5p and reduced FNDC5 in the VSMCs of SHR contribute to enhanced oxidative stress and cell migration.

## 1. Introduction

Vascular smooth muscle cells (VSMCs) are the primary cells in the media of arteries. VSMC migration plays a crucial role in the pathogenesis of several vascular diseases such as hypertension, atherosclerosis, and vascular injury [1]. It involves not only the maladaptive vascular remodeling in hypertension but also the atherogenic mechanisms of hypertension [2]. Angiotensin (Ang) II induces oxidative stress, cell migration, and proliferation in VSMCs [3,4]. Oxidative stress is closely associated with VSMC migration, proliferation, and inflammation in relation to the vascular remodeling of hypertension [4,5,6]. The major source of reactive oxygen species (ROS) in arteries is the NADPH oxidase (NOX) family [7]. Of the NOX isoforms, NOX2 has the greatest implication in vascular diseases. The upregulation of NOX2 induces oxidative stress, and the inhibition of NOX2 reduces ROS production in vascular diseases [8]. Oxidative stress-related biomarkers may have a potential prognostic value for vascular diseases [9]. The inhibition of oxidative stress is considered to be a therapeutic strategy for cardiovascular diseases including hypertension [10].

Fibronectin type III domain containing 5 (FNDC5) is a transmembrane protein with a short cytoplasmic domain, a transmembrane domain, and a fibronectin type III domain [11,12]. Our previous studies showed that FNDC5 overexpression and deficiency, respectively, attenuate and aggravate high fat diet-induced hyperlipemia, hepatic lipid accumulation, and impaired fatty acid β-oxidation (FAO) and autophagy in the liver [13]. Irisin, a myokine cleaved from FNDC5, was found to attenuate insulin resistance and metabolic abnormalities in diabetic mice [14]. FNDC5 promoted lipolysis in the adipose tissues of obese mice [15], and it attenuated adipose tissue inflammation in HFD-induced obese mice [16]. FNDC5 inhibits oxidized low-density lipoprotein (oxLDL)-induced monocyte adhesion and foam cell formation in VSMCs [17], and it attenuates angiotensin II-induced oxidative stress and NLRP3 inflammasome activation in VSMCs [18].

MicroRNAs (miRNAs) are a class of endogenous, non-coding small RNAs (about 18–22 nucleotides) that negatively regulate gene expression by binding to the mRNAs of target genes, specifically on a posttranscriptional level [19]. The dysregulation of miRNAs contributes to the pathogenesis of oxidative stress [20,21], inflammation [22], and several diseases such as cancer [23] and vascular diseases [24,25]. miR-31-5p inhibits the cell migration of renal cell carcinoma [26] and hepatocellular carcinoma [27], but it promotes cell migration in colorectal cancer cells [28]. Moreover, miR-31 was found to promote the proliferation and migration of human VSMCs pretreated with platelet-derived growth factor-BB (PDGF-BB) by targeting mitofusin-2 [29]. However, it is unknown whether miR-31-5p is involved in oxidative stress. The role of miR-31-5p in VSMC migration remains unknown. The authors of the present study aimed to investigate the role of miR-31-5p and its relation to FNDC5 in the oxidative stress and VSMC migration in hypertension.

## 2. Materials and Methods

### 2.1. Cell Culture

Eight-week-old male Wistar–Kyoto rats (WKY; *n* = 9) and spontaneously hypertensive rats (SHR; *n* = 9) were obtained from Vital River Laboratory Animal Technology Co. Ltd. (Beijing, China) and used for isolating primary rat aortic VSMCs. For Western blot and RT-PCR tests, the samples were randomly obtained from 3 WKY-derived VSMCs and 3 SHR-derived VSMCs. For other measurements, the samples were randomly obtained from 6 WKY-derived VSMCs and 6 SHR-derived VSMCs. Commercial rat thoracic aortic smooth muscle cells (A7r5 cells) were obtained from ATCC (Manassas, VA, USA). The experiments were approved by the Experimental Animal Care and Use Committee of Nanjing Medical University and conformed to the Guide for the Care and Use of Laboratory Animal published by the US National Institutes of Health (NIH publication, 8th edition, 2011). The VSMC culture was conducted as we previously reported [24,30]. The rat was anesthetized with pentobarbital sodium (60 mg/kg; ip). Then, the thoracic aorta was excised, and the adventitia and intima were stripped off. The aortic media were treated with 0.2% type 1A collagenase in PBS at 37 °C for 15 min for digestion, and the suspension was centrifuged at 500 × g for 10 min for the isolation of VSMCs. The isolated VSMCs were cultured in Dulbecco’s Modified Eagle Medium (DMEM, Invitrogen, Carlsbad, CA, USA) with 10% fetal bovine serum (FBS), 100 IU/mL of penicillin, and 10 mg/mL of streptomycin in a 37 °C, 5% CO_2_, humidified incubator. The VSMCs between the second and the sixth passages were used in the present study.

### 2.2. Determination of VSMC Migration

VSMC migration was evaluated with both the Boyden chamber and wound healing assays [31]. For the Boyden chamber assay, VSMCs at a density of 5 × 10^4^ cells/mL were seeded onto the upper surface of the 24-well transwell cell culture chambers with 8 μm sized pores (Merck kGaA, Darmstadt, Germany) in serum-free media, and a 10% FBS medium was added to the lower chamber. After 24 h of incubation, the non-migrating VSMCs on the upper surface of the membrane were gently scraped off with cotton swabs, and the migrated VSMCs on the lower surface of the filter were stained with 1% crystal violet and photographed with an inverted microscope in five randomly selected visual fields in each chamber. The average number of the migrated cells was counted from six independent samples in each group. For the wound-healing assay, VSMCs at a density of 2 × 10^5^ cells/mL were seeded in six-well plates and cultured for additional 24 h until they reached about 80–90% confluence. The near-confluent VSMCs in plates were wounded by scraping with a standard 1 mL pipette tip to make a gap in the central region. Then, cellular debris was washed with PBS and a fresh medium was added. The wound healing was monitored and photographed at 0 and 24 h with an inverted microscope (Axio Vert. A1, Zeiss, Oberkochen, Germany). The migrated distances were calculated with the following formula: migrated distance (μm) = (average gap width at 0 h − average gap width at 24 h)/2.

### 2.3. Transfection of miR-31-5p Mimic and Inhibitor

An RNAifectin™ transfection reagent was obtained from Invitrogen (Shanghai, China). Scrambled sequences of the miR-31-5p mimic and inhibitor were used as negative controls to exclude the impact of the reagent or transfection protocol on the target protein. The commercial miR-31-5p mimic and inhibitor, as well as their negative controls (NCs), were purchased from RiboBio (Guangzhou, China). The VSMCs at a density of 2 × 10^5^ cells/mL in 6-well plates were cultured for 24 h until they reached about 80–90% confluence. PBS, NC, miR-31-5p mimic (50 nmol/L) or inhibitor (100 nmol/L), and the RNAifectin™ transfection reagent (6 µL) were simultaneously added into the medium for 6 h. The medium was replaced to remove the transfection reagent. Measurements were carried out 24 h after transfection [24]. The concentration of the miR-31-5p mimic or inhibitor used in the present study was determined according to the manufacturer’s product instructions and our preliminary study. This concentration was also used by the most of the previous studies.

### 2.4. Luciferase Reporter Assay

Luciferase reporter plasmids, pcDNA-FNDC5 and pcDNA-FNDC5-mut, were designed and constructed by Generay Biotech Co., Ltd. (Shanghai, China). For the luciferase reporter assay, VSMCs were seeded and co-transfected with 1 μg/mL of pcDNA-FNDC5-WT or pcDNA-FNDC5-mut reporter plasmids, followed with PBS, normal control (NC) and the miR-31-5p mimic (50 nmol/L) using the Lipofectamine™ 3000 transfection reagent. Luciferase activity was calculated by the dual-luciferase reporter assay system.

### 2.5. Western Blot Analysis

Samples were homogenized in an ice-cold RIPA lysis buffer containing 1% PMSF (Beyotime Biotechnology, Shanghai, China). Protein extracts were electrophoresed, blotted, and incubated with corresponding primary antibody for 24 h, and then incubated with secondary HRP-conjugated antibody for 2 h. The bands were detected with an Enhanced Chemiluminescence Detection Kit (Thermo Scientific, Rockford, IL, USA). The relative values of protein expression were normalized by β-actin. Antibodies against FNDC5 (ab174833, 1:1000), NADPH oxidase 2 (NOX2, ab129068, 1:1000), and β-actin (ab6276, 1:5000) were obtained from Abcam (Cambridge, MA, USA). Antibodies against NOX4 (14347-1-AP, 1:1000) were purchased from Protein Tech Group Inc. (Chicago, IL, USA).

### 2.6. RT-PCR

Total RNA was exacted with a Trizol reagent (Life Technologies, Gaithersburg, MD, USA). Reverse transcriptase reactions were conducted using the PrimeScript RT reagent Kits. RT-PCR was performed using Quantitative PCR with SYBR Premix Ex Taq TM (Takara, Otsu, Shiga, Japan) and the ABI PRISM 7500 sequence detection PCR system (Applied Biosystems, Foster City, CA, USA). The mRNA levels were normalized to β-actin. Primers of FNDC5 and β-actin were obtained from Invitrogen (Shanghai, China), and the primer of FNDC5 was chosen in reference to a previous study in which the primer sequences were designed using the NCBI primer design tool [32]. RT-PCR for miR-31-5p was measured with a commercial kit containing primers for miR-31-5p and U6 (TIANGEN Biotech, Beijing, China). The primers for miR-31-5p and U6 were designed and identified by Tiangen Biotech Co. Ltd. The miR-31-5p levels were normalized to U6. The primers were found to be compliant with MIQE guidelines [33] based on their Ct values determined with standard curve, and the sequences of primers are indicated in Table 1. 

### 2.7. DHE Fluorescence Staining

The ROS level in VSMCs was evaluated with dihydroethidium (DHE) fluorescence staining. The VSMCs (about 3 × 10^5^ cells/mL) were incubated in six-well plates with PBS containing DHE (10 μM) in a dark and humidified container at 37 °C for 30 min, and then the cell nuclei were stained with 4′,6-diamidino-2-phenylindole (DAPI) at room temperature for 10 min. After being washed three times with PBS, the DHE fluorescence was photographed under excitation at 518 nm and emission at 605 nm with a fluorescence microscope (DP70, Olympus Optical, Tokyo, Japan). The fluorescent intensities of the DHE-labeled VSMCs were quantified with ImageJ 1.8.0. (Wayne Rsband, NIH, USA).

### 2.8. Evaluation of Cell Viability

Cell viability was examined with a CCK8 kit (Beyotime Biotechnology, Shanghai, China) following the manufacturer’s instructions. The CCK8 kit is widely used for cell proliferation and cytotoxicity assays. The absorbance was measured with a microplate reader (Model ELX800, BioTek, Winooski, VT, USA) at 450 nm [24].

### 2.9. Statistical Analysis

Experiments were carried out in a double-blinded and randomized fashion. Comparisons between two groups were made by Student’s t-test. One-way or two-way ANOVA was used for multiple comparisons, followed by the post hoc Bonferroni’s test. All data are expressed as mean ± SE. A *p* value of less than 0.05 was considered statistically significant.

## 3. Results

### 3.1. Roles of miR-31-5p in Oxidative Stress

**Figure 1:**
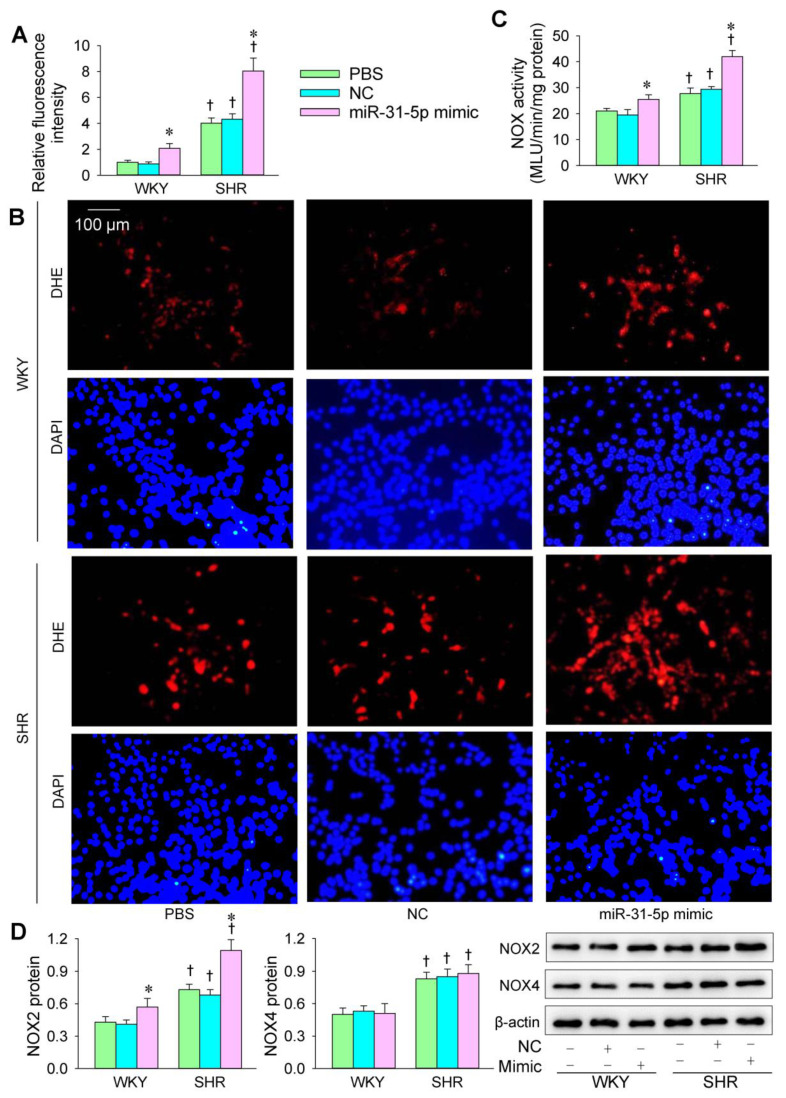
Effects of the miR-31-5p mimic on oxidative stress in VSMCs of WKY and SHR. The measurements were made after treatment with PBS, normal control (NC, 50 nmol/L), or miR-31-5p mimic (50 nmol/L) for 24 h. (**A**,**B**) ROS production was detected by dihydroethidium (DHE) fluorescent staining (red), and cell nuclei were stained with DAPI (blue). (**C**) NOX activity. (**D**) NOX2 and NOX4 protein expression. Values are mean ± SE. * *p* < 0.05 vs. PBS or NC; † *p* < 0.05 vs. WKY. *n* = 6 per group in (**A**,**C**), and *n* = 3 per group in (**D**).

**Figure 2:**
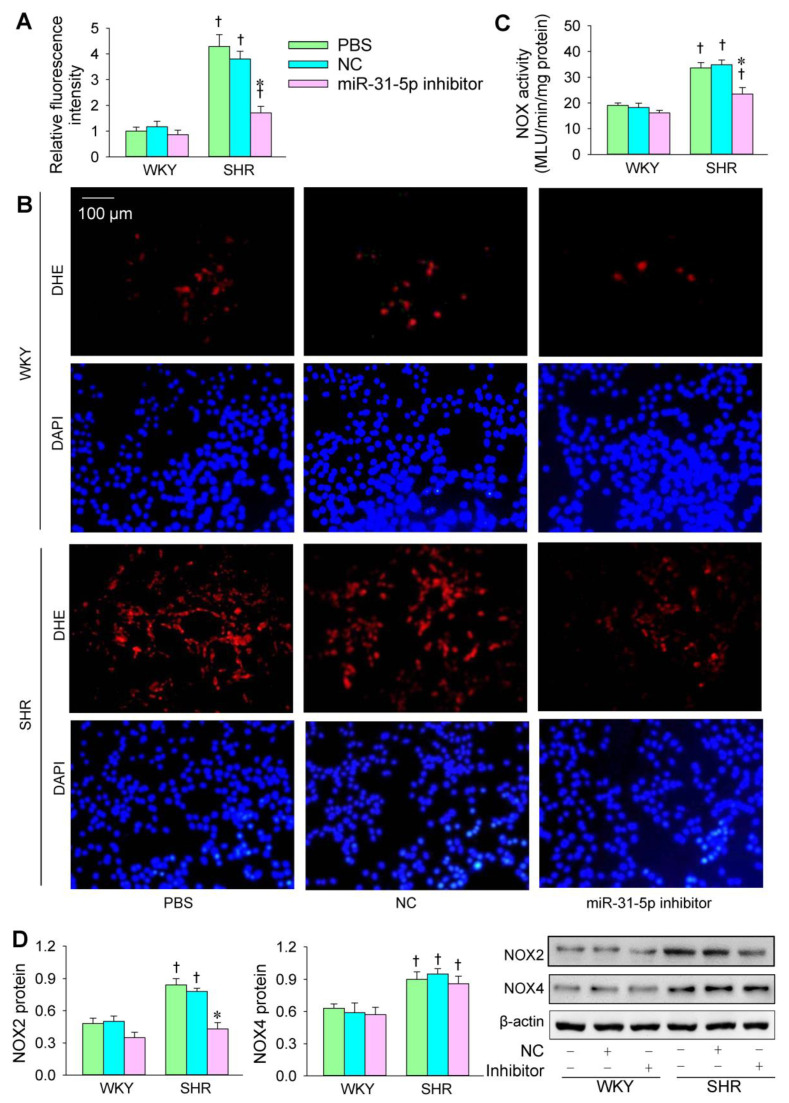
Effects of the miR-31-5p inhibitor on oxidative stress in VSMCs of WKY and SHR. The measurements were made after treatment with PBS, normal control (NC, 100 nmol/L), or miR-31-5p inhibitor (100 nmol/L) for 24 h. (**A**,**B**) ROS production was detected by dihydroethidium (DHE) fluorescent staining (red), and cell nuclei were stained with DAPI (blue). (**C**) NOX activity. (**D**) NOX2 and NOX4 protein expression. Values are mean ± SE. * *p* < 0.05 vs. PBS or NC; † *p* < 0.05 vs. WKY. *n* = 6 per group in (**A**,**C**); *n* = 3 per group in (**D**).

The transfection of the miR-31-5p mimic or inhibitor was carried out to determine the roles of miR-31-5p in oxidative stress in VSMCs of WKY and SHR. The ROS production in the VSMCs was greater in SHR than in WKY. The miR-31-5p mimic increased ROS production and NOX activity in both the WKY and SHR (Figure 1A–C). Both NOX2 and NOX4 protein expression in VSMCs was increased in the SHR. The miR-31-5p mimic increased NOX2 expression but not NOX4 expression (Figure 1D). The miR-31-5p inhibitor reduced ROS production and NOX activity in the VSMCs of the SHR but not those of the WKY (Figure 2A–C). Furthermore, the miR-31-5p inhibitor normalized NOX2 expression in the SHR but had no significant effects on NOX4 expression (Figure 2D). These results indicate that miR-31-5p promotes oxidative stress and the inhibition of endogenous miR-31-5p attenuates oxidative stress in the VSMCs of SHR.

### 3.2. Roles of miR-31-5p in VSMC Migration

**Figure 3:**
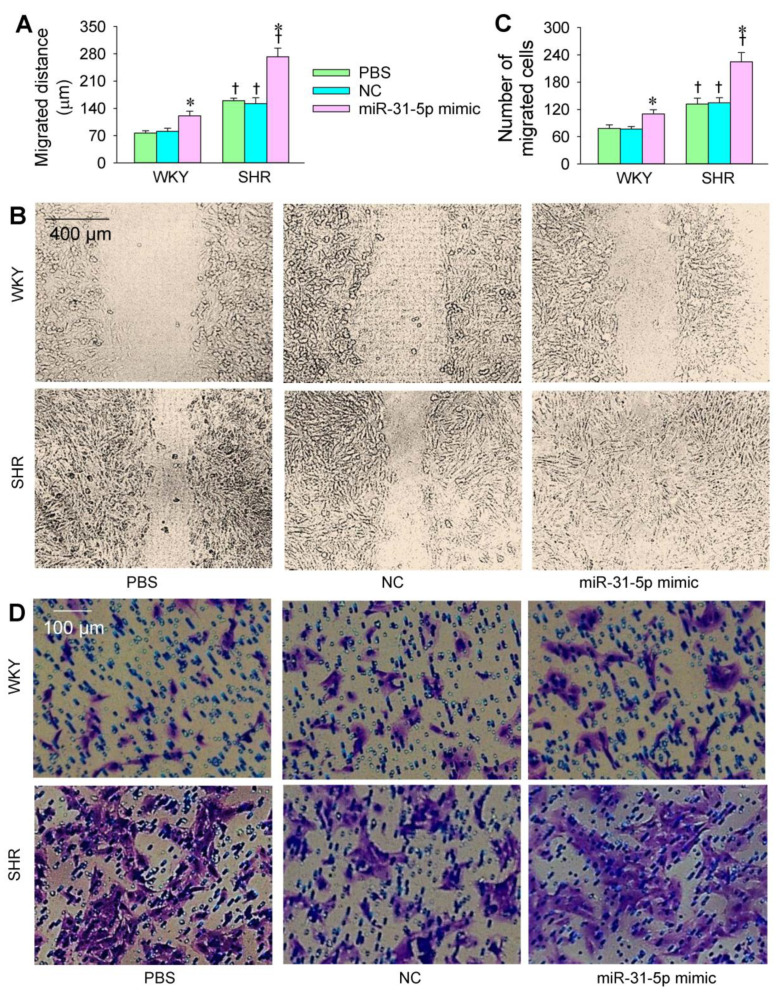
Effects of the miR-31-5p mimic on the VSMC migration of WKY and SHR. The measurements were made after treatment with PBS, normal control (NC, 50 nmol/L), or miR-31-5p mimic (50 nmol/L) for 24 h. (**A**,**B**) VSMC migration was determined with a wound healing assay; (**C**,**D**) VSMC migration was evaluated with a Boyden chamber assay. Values are mean ± SE. * *p* < 0.05 vs. PBS or NC; † *p* < 0.05 vs. WKY. *n* = 6 per group.

**Figure 4:**
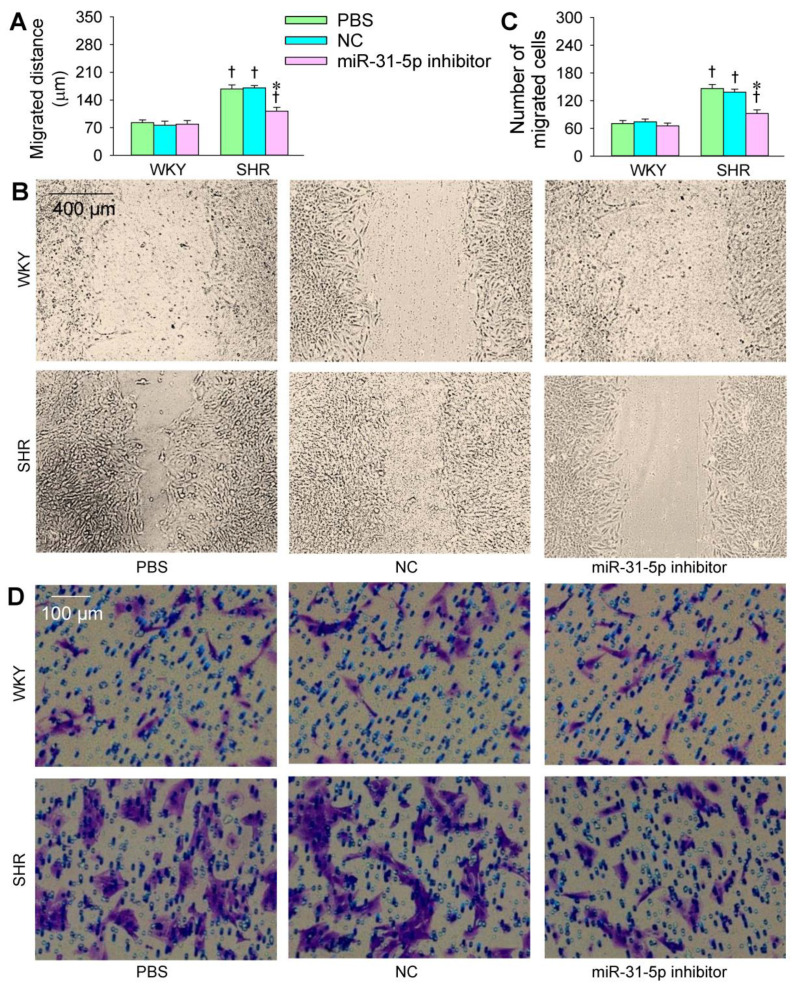
Effects of the miR-31-5p inhibitor on the VSMC migration of WKY and SHR. The measurements were made after treatment with PBS, normal control (NC, 100 nmol/L), or miR-31-5p inhibitor (100 nmol/L) for 24 h. (**A**,**B**) VSMC migration was determined with a wound healing assay; (**C**,**D**) VSMC migration was evaluated with a Boyden chamber assay. Values are mean ± SE. * *p* < 0.05 vs. PBS or NC; † *p* < 0.05 vs. WKY. *n* = 6 per group.

The transfection of the miR-31-5p mimic or inhibitor was carried out to determine the roles of miR-31-5p in the VSMC migration of WKY and SHR. The VSMC migration was evaluated with wound healing and Boyden chamber assays. VSMC migration was enhanced in the SHR compared with that in the WKY. The miR-31-5p mimic promoted the VSMC migration of both the WKY and SHR (Figure 3A–D), while the miR-31-5p inhibitor attenuated the VSMC migration in the SHR but not in the WKY (Figure 4A–D). On the other hand, both the miR-31-5p mimic and inhibitor had no significant cytotoxic effects on the VSMCs of the WKY and SHR (Figure S1 in the online supplement).

### 3.3. FNDC5 Is a Target of miR-31-5p

**Figure 5:**
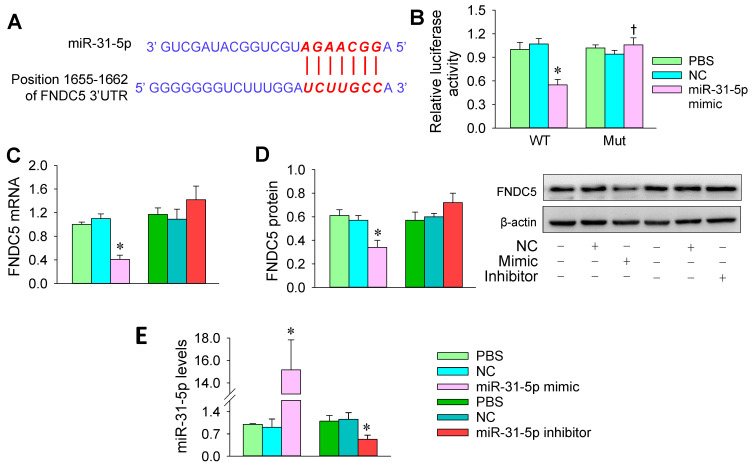
FNDC5 is one of the targets of miR-31-5p in A7r5 cells. (**A**) Predicted location of the miR-31-5p combination according to TargetScanHuman. (**B**) Relative luciferase activity in A7r5 cells. The cells were transfected with pcDNA-FNDC5-WT or pcDNA FNDC5-mut plasmids, followed by PBS, normal control (NC, 50 nmol/L), or miR-31-5p mimic (50 nmol/L) treatment for 24 h. (**C**) Effects of the miR-31-5p mimic and inhibitor on FNDC5 mRNA expression. (**D**) Effects of the miR-31-5p mimic and inhibitor on FNDC5 protein expression. (**E**) Effects of the miR-31-5p mimic and inhibitor on miR-31-5p levels. Measurements were performed after treatment with PBS, normal control (NC, 50 nmol/L), miR-31-5p mimic (50 nmol/L), or miR-31-5p inhibitor (100 nmol/L) for 24 h. Values are mean ± SE. * *p* < 0.05 vs. PBS or NC; † *p* < 0.05 vs. WT. *n* = 3 per group.

miR-31-5p is involved in the function of vascular endothelial cells via targeting ETBR, Notch 1, or eNOS [34,35,36], but little is known about the roles and targets of miR-31-5p in the VSMCs of hypertension. TargetScanHuman (http://www.targetscan.org/; accessed on 21 March 2020), an online predictive tool for miRNA targets in mammals, suggests that FNDC5 might be a target of miR-31-5p. The location of this targeting is predicted to be 1655-1662 of FNDC5 3’ -UTR (Figure 5A). To verify this prediction, luciferase reporter plasmids containing wild-type (WT) or mutant FNDC5 (Mut) were constructed and transfected to A7r5 cells, a rat thoracic aortic smooth muscle cell line. Relative luciferase assays showed that the miR-31-5p mimic inhibited the activity of luciferase-reporter-harboring wild-type FNDC5 but not mutant FNDC5 (Figure 5B). The results were supported by the finding that the miR-31-5p mimic significantly inhibited FNDC5 mRNA and protein expression in A7r5 cells. However, the miR-31-5p inhibitor did not significantly promote FNDC5 expression in these cells (Figure 5C,D). The effectiveness of the transfection of the miR-31-5p mimic and inhibitor was confirmed by the corresponding changes of miR-31-5p levels in A7r5 cells (Figure 5E).

### 3.4. miR-31-5p Levels and the Roles of miR-31-5p in Regulating FNDC5 Expression

**Figure 6:**
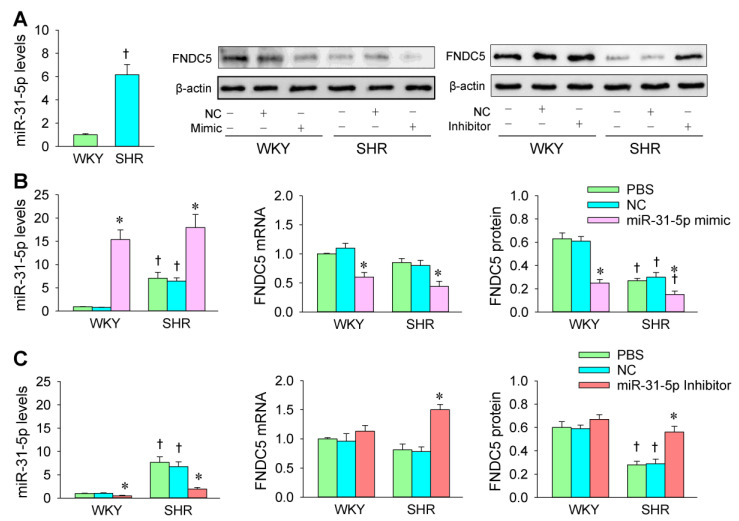
Levels of miR-31-5p and effects of the miR-31-5p mimic and inhibitor on the FNDC5 expression in the VSMCs of WKY and SHR. (**A**) miR-31-5p levels in the VSMCs. (**B**) Effects of the miR-31-5p mimic on miR-31-5p, FNDC5 mRNA, and protein expression in VSMCs. (**C**) Effects of the miR-31-5p inhibitor on miR-31-5p, FNDC5 mRNA, and protein expression in VSMCs. The measurements were performed after treatment with PBS, normal control (NC, 50 nmol/L), miR-31-5p mimic (50 nmol/L), or miR-31-5p inhibitor (100 nmol/L) for 24 h. Values are mean ± SE. * *p* < 0.05 vs. PBS or NC; † *p* < 0.05 vs. WKY. *n* = 3 per group.

We compared the difference of miR-31-5p levels between WKY and SHR. The miR-31-5p levels were much higher in the VSMCs of the SHR than those in the WKY (Figure 6A), suggesting that the increased miR-31-5p in the VSMCs of SHR might be involved in the enhanced oxidative stress and VSMC migration in hypertension. In order to determine whether miR-31-5p is involved in the regulation of FNDC5 expression in hypertension, the effects of the miR-31-5p mimic and inhibitor on FNDC5 expression were further identified in primary VSMCs of WKY and SHR. The effectiveness of the transfection of the miR-31-5p mimic and inhibitor was confirmed by the changes of miR-31-5p levels and their target protein FNDC5 expression in VSMCs (Figure 6B,C). The FNDC5 protein expression in the VSMCs of SHR was less than that of WKY. The miR-31-5p mimic reduced FNDC5 mRNA and protein expression in the VSMCs of both WKY and SHR (Figure 6B). However, the miR-31-5p inhibitor only increased FNDC5 expression in the VSMCs of SHR, not WKY (Figure 6C). These findings indicate that miR-31-5p inhibits FNDC5 expression in the VSMCs of WKY and SHR.

### 3.5. Effects of Exogenous FNDC5 on Oxidative Stress and VSMC Migration

**Figure 7:**
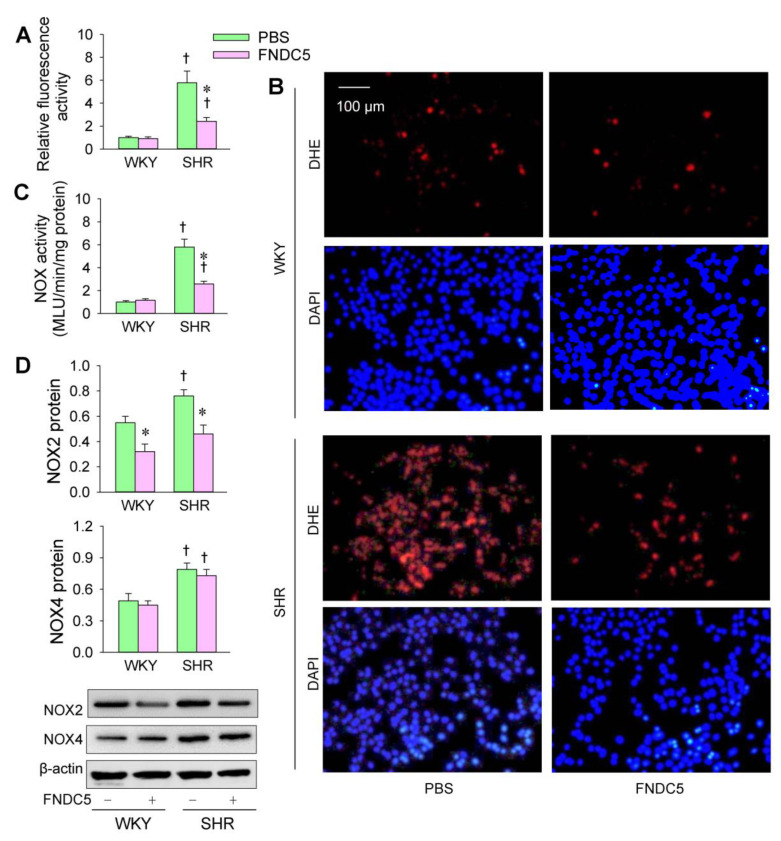
Effects of exogenous FNDC5 on oxidative stress in the VSMCs of WKY and SHR. The measurements were made after treatment with PBS or FNDC5 (200 nmol/L) for 24 h. (**A**,**B**) The effects of FNDC5 on ROS production were detected by dihydroethidium (DHE) fluorescent staining (red), and cell nuclei were stained with DAPI (blue). (**C**) Effects of FNDC5 on NOX activity. (**D**) Effects of FNDC5 on NOX2 and NOX4 expression. Values are mean ± SE. * *p* < 0.05 vs. PBS; † *p* < 0.05 vs. WKY. *n* = 6 per group in (**A**–**C**); *n* = 3 per group in (**D**).

**Figure 8:**
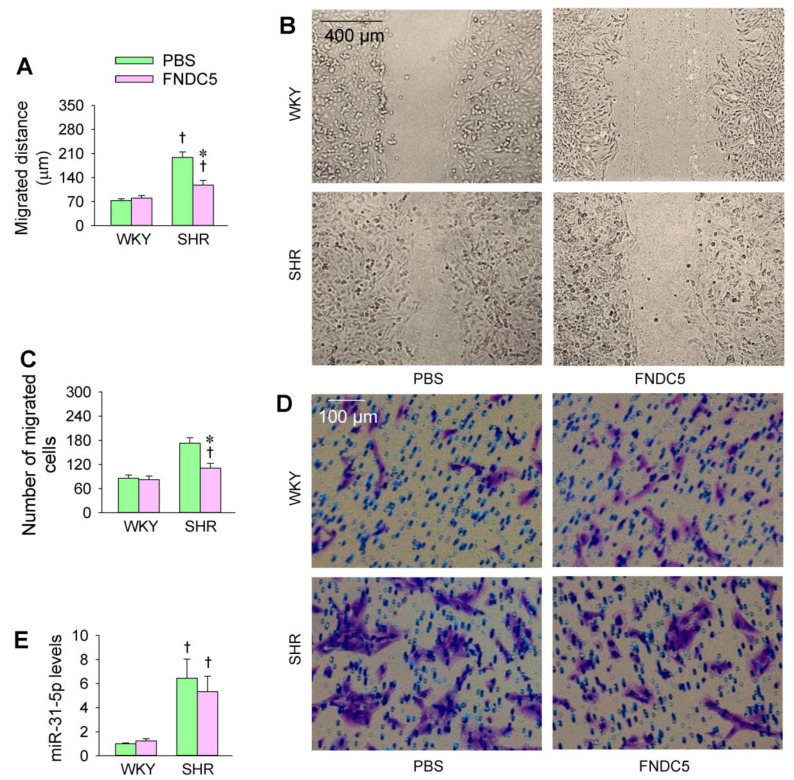
Effects of exogenous FNDC5 on VSMC migration and miR-31-5p levels in WKY and SHR. The measurements were made after treatment with PBS or FNDC5 (200 nmol/L) for 24 h. (**A**,**B**) VSMC migration was evaluated with a wound healing assay. (**C**,**D**) VSMC migration was determined with a Boyden chamber assay. (**E**) miR-31-5p levels. Values are mean ± SE. * *p* < 0.05 vs. PBS; † *p* < 0.05 vs. WKY. *n* = 6 per group.

An interesting question is whether endogenous FNDC5 could prevent enhanced oxidative stress and VSMC migration in SHR. We found that the application of FNDC5 (Sigma Inc., St Louis, MO, USA) reduced ROS production and NOS activity in SHR (Figure 7A–C). The endogenous FNDC5 inhibited NOX2 expression in the VSMCs of both the WKY and SHR but had no significant effects on NOX4 expression (Figure 7D). Moreover, FNDC5 inhibited cell migration in the VSMCs of the SHR but not WKY (Figure 8A–D). These results indicate that FNDC5 attenuates oxidative stress and VSMC migration in SHR. On the other hand, FNDC5 had no significant effects on miR-31-5p level in the VSMCs of both the WKY and SHR (Figure 8E).

### 3.6. FNDC5 Prevents miR-31-5p Mimic-Induced Oxidative Stress and VSMC Migration

**Figure 9:**
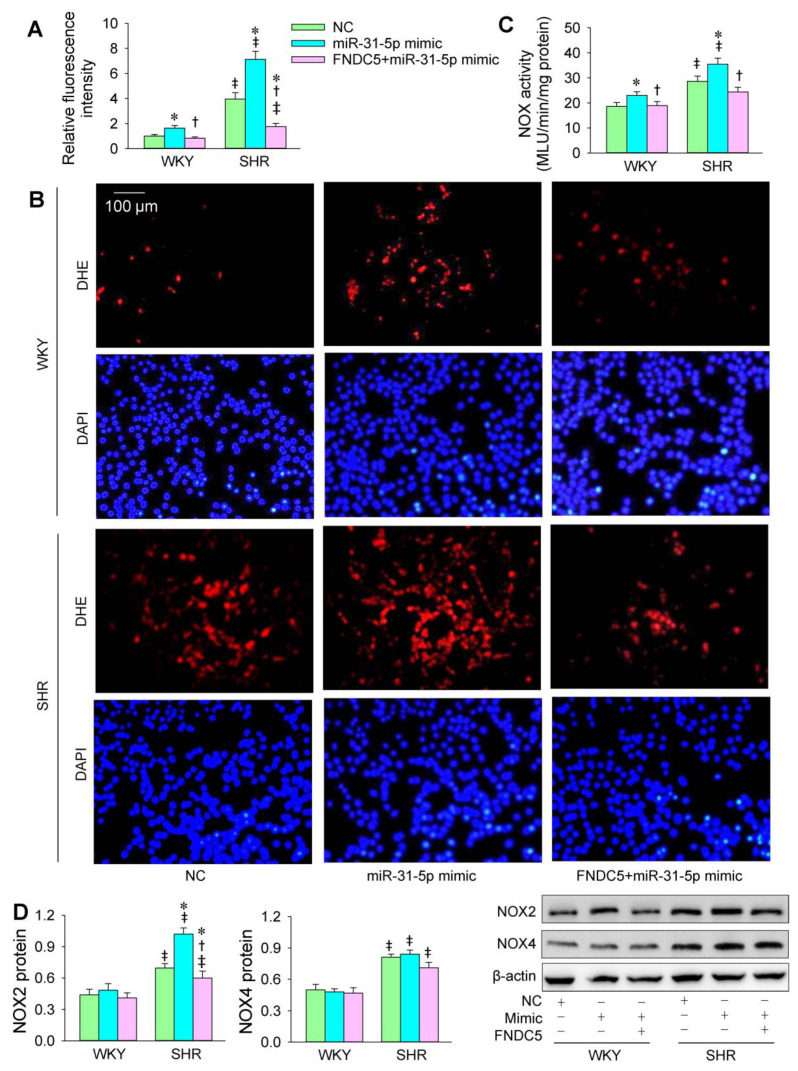
Effects of exogenous FNDC5 on miR-31-5p mimic-induced oxidative stress in VSMCs of WKY and SHR. The measurements were made after treatment with normal control (NC, 50 nmol/L), miR-31-5p mimic (50 nmol/L), or FNDC5 (200 nmol/L) and miR-31-5p mimic (50 nmol/L) for 24 h. (**A**,**B**) ROS production was detected by dihydroethidium (DHE) fluorescent staining (red), and cell nuclei were stained with DAPI (blue). (**C**) NOX activity. (**D**) NOX2 and NOX4 protein expression. Values are mean ± SE. * *p* < 0.05 vs. NC; † *p* < 0.05 vs. miR-31-5p; ‡ *p* < 0.05 vs. WKY. *n* = 6 per group in (**A**–**C**); *n* = 3 per group in (**D**).

**Figure 10:**
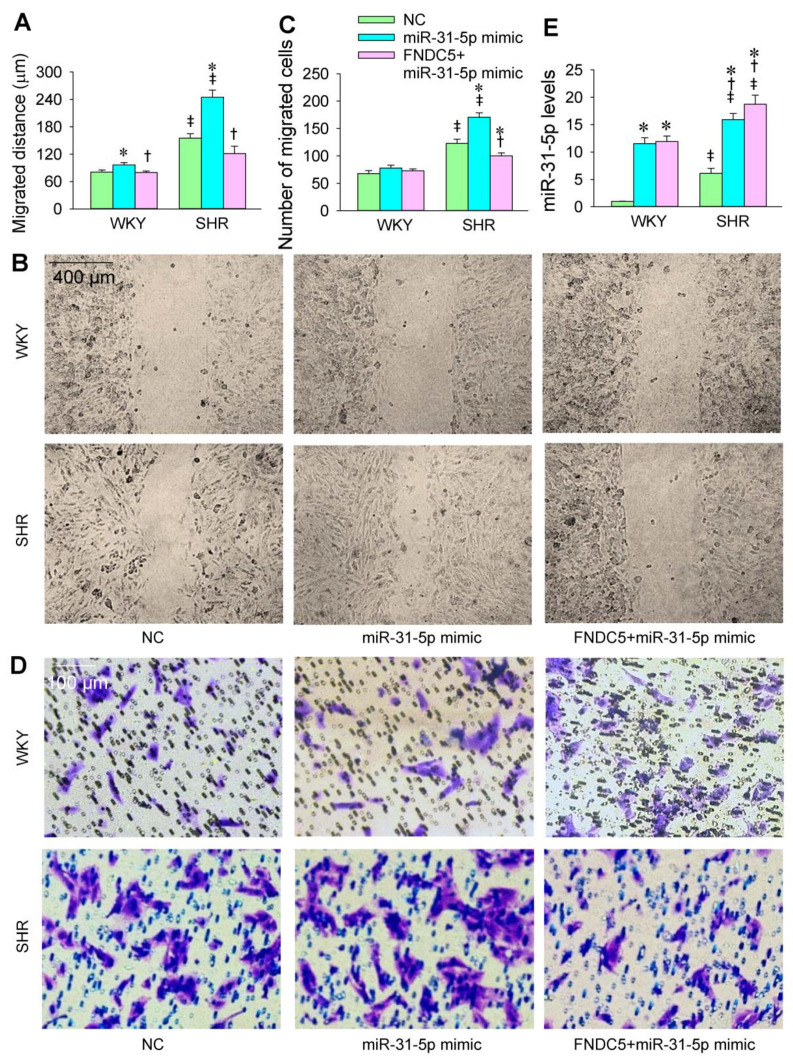
Effects of exogenous FNDC5 on miR-31-5p mimic-induced VSMC migration in WKY and SHR. The measurements were made after treatment with normal control (NC, 50 nmol/L), miR-31-5p mimic (50 nmol/L), or FNDC5 (200 nmol/L) and miR-31-5p mimic (50 nmol/L) for 24 h. (**A**,**B**) VSMC migration was determined with wound healing assay. (**C**,**D**) VSMC migration was determined with Boyden chamber assay. (**E**) miR-31-5p levels. Values are mean ± SE. * *p* < 0.05 vs. NC; † *p* < 0.05 vs. miR-31-5p; ‡ *p* < 0.05 vs. WKY. *n* = 6 per group.

In order to provide further evidence that the roles of miR-31-5p are mediated by inhibiting FNDC5 expression, we examined the roles of exogenous FNDC5 in attenuating miR-31-5p mimic-induced oxidative stress and VSMC migration. The application of exogenous FNDC5 inhibited the roles of the miR-31-5p mimic in promoting ROS production, NOX activity, and NOX2 expression (Figure 9A–D). Similarly, FNDC5 attenuated the roles of the miR-31-5p mimic in promoting VSMC migration (Figure 10A–D). FNDC5 had no significant effect on miR-31-5p levels (Figure 10E). These results provide further evidence that miR-31-5p promotes oxidative stress and VSMC migration via inhibiting FNDC5.

## 4. Discussion

Oxidative stress is closely associated with vascular remodeling in hypertension [10,37]. The primary novel findings of this study are that miR-31-5p promotes oxidative stress and cell migration in the VSMCs of SHR by reducing FNDC5 expression and that the inhibition of endogenous miR-31-5p attenuates oxidative stress and VSMC migration in SHR by normalizing FNDC5 expression. The increased miR-31-5p level in the VSMCs of SHR at least partially contributes to the oxidative stress and VSMC migration in SHR.

The inhibition of endogenous miR-31-5p attenuates VSMC migration in SHR, which may play beneficial roles in attenuating the maladaptive vascular remodeling in SHR. VSMC migration was found to be enhanced in SHR, which is supported by the results of previous studies [38,39,40]. Though VSMC migration into already populated intima or even after cellular destruction is very slow [41], it contributes to hypertension-induced maladaptive vascular remodeling [42]. Moreover, enhanced VSMC migration contributes to the atherogenic mechanisms and has implications for the atherogenesis of hypertension [2,43]. It is noted that microRNAs could mimic phenotypes induced by mutations of the protein completely unrelated to this phenotype [44]. Moreover, extracellular matrix and intercellular adhesion affect the VSMC migration process. It is possible that miR-31-5p may affect extracellular matrix and intercellular adhesion, which consequently promote VSMC migration.

ROS is involved in intracellular signaling pathways in physiological functions as secondary messengers [45]. Chronic vascular oxidative stress contributes to the pathogenesis of hypertension [10]. NOX-derived ROS in the media of the vascular wall is the dominant reason for intracellular redox signaling cascade activation, further leading to the proliferation and migration of blood vessels [8,46,47]. The inhibition of excessive oxidative stress is beneficial for hypertension [8,10]. In the present study, miR-31-5p served as an endogenous oxidant and was found to be associated with the balance between oxidation and anti-oxidation. The roles of miR-31-5p in promoting VSMC migration are partially secondary to its promoting effects on oxidative stress. The inhibition of endogenous miR-31-5p may be used as a therapeutic strategy in attenuating oxidative stress in hypertension.

In contrast to WKY, FNDC5 expression was found to be reduced while the miR-31-5p level was increased in the VSMCs of SHR. The increased miR-31-5p level in the VSMCs of SHR was responsible for the downregulation of FNDC5. FNDC5 inhibited oxidative stress and VSMC migration in the VSMCs of SHR. Most importantly, FNDC5 was found to attenuate the roles of the miR-31-5p mimic in promoting oxidative stress and VSMC migration in the VSMCs of SHR. These findings provide solid evidence that miR-31-5p promotes oxidative stress and VSMC migration by inhibiting FNDC5 expression. The inhibition of endogenous miR-31-5p prevents FNDC5 downregulation and therefore attenuates oxidative stress and VSMC migration in SHR. It is known that FNDC5 attenuates insulin resistance and glucose and lipid metabolism disorders [13,14,15]. We speculate that inhibiting miR-31-5p to increase FNDC5 may be a therapeutic strategy with some unique advantages in oxidative stress attenuation, vascular remodeling, and hypertension with obesity, diabetes, and atherosclerosis.

Our recent study showed that FNDC5 deficiency and application, respectively, aggravates and attenuates Ang II-induced NOX2 upregulation and NLRP3 inflammasome activation via the AMPK- SIRT1 signal pathway in VSMCs [18]. The roles of miR-31-5p in promoting oxidative stress and VSMC migration may be attributed to FNDC5 downregulation in both WKY and SHR. The level of miR-31-5p is greatly increased in the VSMCs of SHR, which causes FNDC5 downregulation. Therefore, the inhibition of endogenous miR-31-5p normalizes the reduced FNDC5 expression and thereby attenuates oxidative stress in SHR. However, a lower miR-31-5p level in the VSMCs of WKY does not play a significant role in inhibiting FNDC5 expression and therefore has no significant effects on oxidative stress and VSMC migration in WKY. On the other hand, an increase in medial smooth muscle herniae into the intima (myointimal herniae) was found in rats with kidney-dependent hypertension [48]. VSMC migration contributes to hypertension-induced maladaptive vascular remodeling [42].

It is interesting that the miR-31-5p mimic inhibits FNDC5 expression in the VSMCs of both WKY and SHR, but the oxidative stress and VSMC migration effects are much greater in SHR than in WKY. It is possible that some compensatory mechanism may prevent oxidative stress and VSMC migration in the VSMCs of WKY, while the compensatory mechanism may be damaged in the VSMCs of SHR. Though FNDC5 expression is reduced in the VSMCs of SHR, it still plays a role in inhibiting oxidative stress and VSMC migration. On the other hand, FNDC5 was found to inhibit NOX2 protein expression but had no significant effect on ROS production in the VSMCs of WKY. We speculate that the reduced ROS production due to the FNDC5-induced NOX2 downregulation is compensated for by other enzymes that promote ROS production, because proper ROS production in cells is necessary for normal signal transduction.

In conclusion, miR-31-5p promotes oxidative stress and VSMC migration in SHR by reducing FNDC5 expression. The inhibition of endogenous miR-31-5p attenuates oxidative stress and VSMC migration in SHR by normalizing FNDC5 expression. The increased miR-31-5p and reduced FNDC5 in the VSMCs of SHR contributes to their oxidative stress and VSMC migration. The intervention of miR-31-5p or the upregulation of FNDC5 may be a potential therapeutic strategy for attenuating oxidative stress in hypertension.

## Figures and Tables

**Table 1:** Primers for real-time quantitative PCR analysis in rats.

Gene	Primer	Sequence (5′ → 3′)
FNDC5	Forward	AGAGAGCAAGCACCAAGACT
	Reverse	GATGGAGTCGGAACCCTGAA
β-actin	Forward	GGACCTGACAGACTACCTCA
	Reverse	GTTGCCAATAGTGATGACCT
miR-31-5p	Forward	GCGCGTGAGATGGCTCCCTG
U6	Forward	CTCGCTTCGGCAGCACA

## Data Availability

The data presented in this study are available on request from the corresponding author on reasonable request.

## Supplementary Materials

The following are available online at https://www.mdpi.com/article/10.3390/biomedicines9081009/s1. Figure S1 Effects of miR-31-5p mimic and inhibitor on VSMC viability of WKY and SHR.
