# Supplementary material for: miR-31-5p Promotes Oxidative Stress and Vascular Smooth Muscle Cell Migration in Spontaneously Hypertensive Rats via Inhibiting FNDC5 Expression
Source: Biomedicines. 2021 Aug 13;9(8):1009. doi: 10.3390/biomedicines9081009 (PMC8393189; doi:10.3390/biomedicines9081009)
Supplement: Supplementary file 1 [file biomedicines-09-01009-s001.zip › biomedicines-1293604-supplementary.pdf]

Online supplemental figure

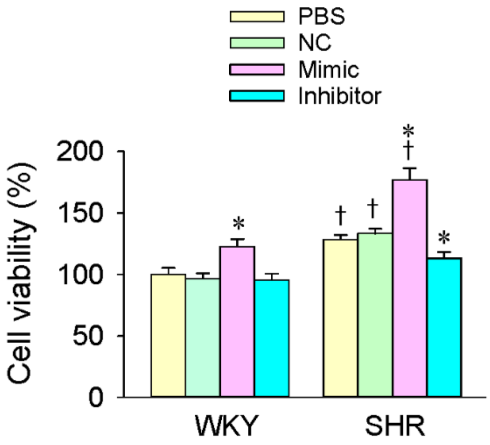

Figure S1 Effects of miR-31-5p mimic and inhibitor on VSMC viability of WKY and SHR. The measurements were made after the treatment with PBS, normal control (NC, 50 nmol/L), miR-31-5p mimic (50 nmol/L) or miR-31-5p inhibitor (100 nmol/L) for 24 h. The cell viability was measured with CCK-8 kits. Values are mean±SE. *$P<0.05$ vs. PBS or NC; †$P<0.05$ vs. WKY. n=6 per group.
